# Myeloid antigen-presenting cells in neurodegenerative diseases: a focus on classical and non-classical MHC molecules

**DOI:** 10.3389/fnins.2024.1488382

**Published:** 2024-12-09

**Authors:** Reham Afify, Katherine Lipsius, Season J. Wyatt-Johnson, Randy R. Brutkiewicz

**Affiliations:** Department of Microbiology and Immunology and Stark Neuroscience Research Institute, Indiana University School of Medicine, Indianapolis, IN, United States

**Keywords:** myeloid cells, microglia, MHC, MR1, CD1d, neurodegenerative diseases

## Abstract

In recent years, increasing evidence has highlighted the critical role of myeloid cells, specifically those that present antigen (APCs) in health and disease. These shape the progression and development of neurodegenerative disorders, where considerable interplay between the immune system and neurons influences the course of disease pathogenesis. Antigen-presenting myeloid cells display different classes of major histocompatibility complex (MHC) and MHC-like proteins on their surface for presenting various types of antigens to a wide variety of T cells. While most studies focus on the role of myeloid MHC class I and II molecules in health and disease, there is still much that remains unknown about non-polymorphic MHC-like molecules such as CD1d and MR1. Thus, in this review, we will summarize the recent findings regarding the contributions of both classical and non-classical MHC molecules, particularly on myeloid microglial APCs, in neurodegenerative diseases. This will offer a better understanding of altered mechanisms that may pave the way for the development of novel therapeutic strategies targeting immune cell-MHC interactions, to mitigate neurodegeneration and its associated pathology.

## Introduction

**Figure 1:**
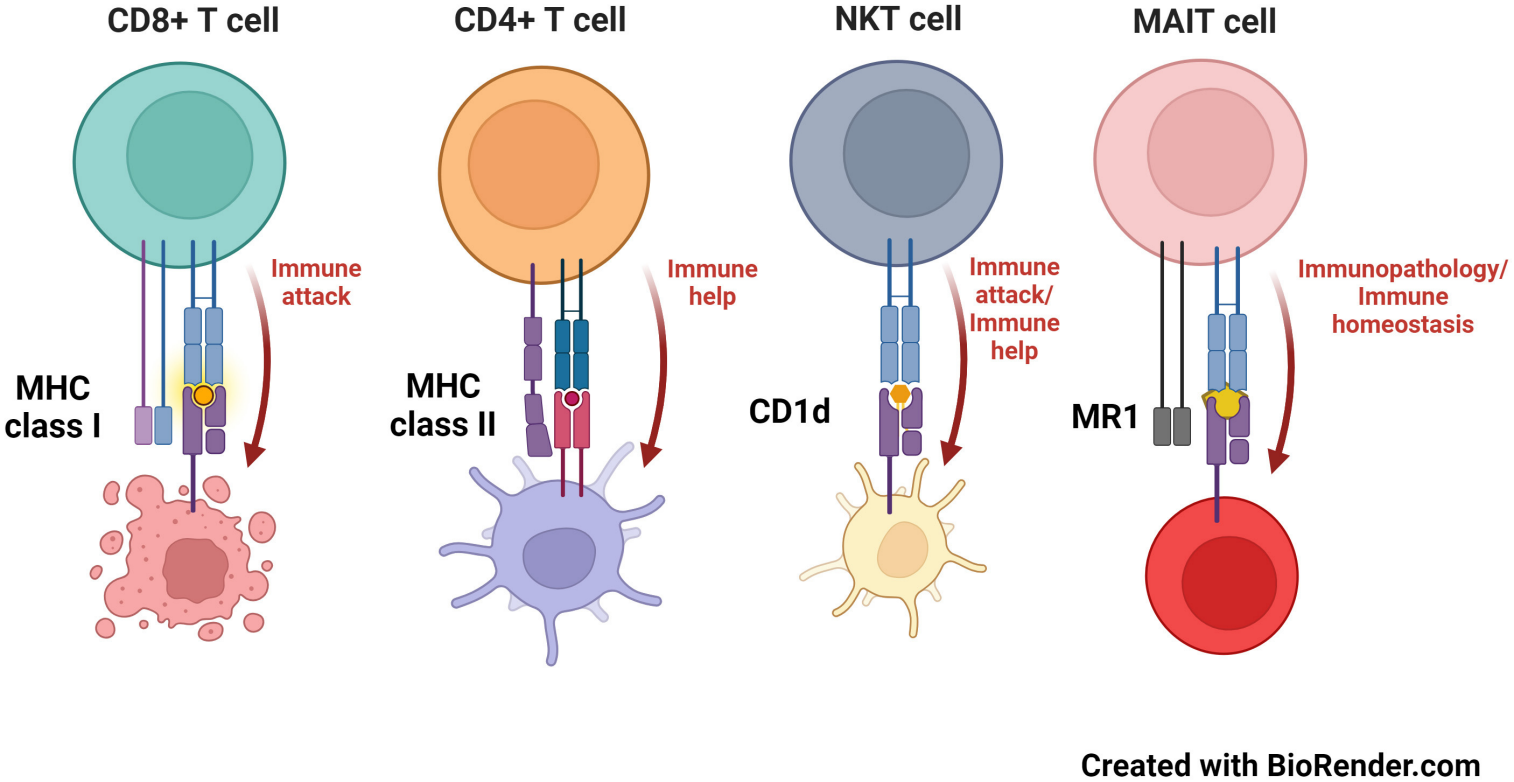
MHC and MHC-like molecules and the T cells that recognize them. MHC class I, MHC class II, CD1d and MR1 molecules present antigens to CD8^+^ (8–9 amino acid peptides), CD4^+^ (peptides that can exceed 20 amino acids in length), NKT (phospho- and glycolipids) and MAIT cells (microbial-derived vitamin B metabolites), respectively.

Recognition of major histocompatibility complex (MHC) molecules is the major means by which mammals (and many other organisms) distinguish between self and non-self (Dausset and Contu, 1980). Classical MHC molecules come in two flavors (Figure 1): MHC class I and MHC class II (Kaufman et al., 1984; Watts and Powis, 1999). These groups present peptides that consist of either 7–9 amino acids (MHC class I) or ~20 or more amino acids (MHC class II), to either CD8^+^ or CD4^+^ T cells, respectively (Swain, 1983; Yewdell and Bennink, 1992; Pishesha et al., 2022). In terms of neuroscience, most cells of the central nervous system (CNS) express very low levels of MHC molecules. As such, an upregulation of MHC class I or class II molecules is viewed that a cell has been activated; thus, measurements of MHC molecules are used as activation markers (Lassmann et al., 1991; Kreutzberg, 1995; Smith, 2001; Hanisch, 2013; Frohman et al., 1989; Fabry et al., 1994; Healy et al., 2020). Whereas detecting an upregulation of MHC molecules can be seen as an activated state in such cells (e.g., MHC class II in epithelial cells), this is usually due to exposure to proinflammatory cytokines, such as IFN-γ, which does indeed induce the expression of MHC molecules during an infection or other inflammatory state (Steinman, 1988; Früh and Yang, 1999; Giacomini et al., 1988).

Related to MHC molecules are those that are called “MHC-like.” These generally have a comparable 3-dimensional structure as classical MHC molecules and many present antigens, although some, like the neonatal Fc receptor, do not (Simister and Ahouse, 1996; Wilson and Bjorkman, 1998). Two main MHC class I-like antigen presenting molecules have been studied in neurobiology—these are called CD1d and MR1 (Figure 1). CD1d presents glycolipids or phospholipids to a population of innate-like T cells named, “natural killer T” (NKT) cells (Brutkiewicz et al., 2018; Bendelac et al., 2007; Brutkiewicz, 2006). NKT cells have the capacity to produce both pro- and anti-inflammatory cytokines, allowing them to help regulate a host's response to a pathogen or cancer (Bendelac et al., 2007; Terabe and Berzofsky, 2014). Moreover, NKT cells rapidly travel into the brain following an ischemic stroke, potentially contributing to the pathology that occurs in such an event (Pan et al., 2021; Wang et al., 2016; Gelderblom et al., 2009; Lehmann et al., 2014), although they have yet to be found/explored in neurodegenerative diseases (Shrinivasan et al., 2024). In contrast, MR1 presents neither peptides nor lipids to T cells. Instead, MR1 presents microbial vitamin B-derived metabolites to another innate-like T cell population called, “mucosal-associated invariant T” (MAIT) cells (Kjer-Nielsen et al., 2012). MAIT cells, as their name suggests, are mainly found in mucosal tissue such as in the lungs or intestine (Birkinshaw et al., 2014; Godfrey et al., 2019; Keller et al., 2017), but have recently been found in the meningeal barrier and brain tissue (Wyatt-Johnson et al., 2023; Zhang et al., 2022). Furthermore, MAIT cells are highly proinflammatory and upon antigen recognition, cause damage to the tissue they reside in, such as that which occurs in inflammatory bowel disease (Ju et al., 2020; Serriari et al., 2014; Tominaga et al., 2017) or in a variety of CNS disorders including neurodegenerative diseases, glioblastoma, cerebral palsy, and ischemia (Salou et al., 2016; Shrinivasan et al., 2024; Wyatt-Johnson et al., 2024).

Immunologists mainly see MHC and MHC-like molecules in their basic function—presenting antigens to T cells. Regardless of the tissue in which these antigen presenting molecules are expressed, they do have the capacity to be recognized by the requisite T cells when the right antigen is presented by the right MHC or MHC-like molecule. Thus, rather than just being activation markers, the classical antigen presenting function of these molecules needs to be considered when studying them in the context of diseases of the CNS. Notably, several myeloid cells in the CNS cells can indeed present antigens to various populations of T cells (Hart and Fabry, 1995; Priya and Brutkiewicz, 2020), illustrating the importance of MHC and MHC-like molecules in CNS disorders. Myeloid cells play a major role in immune surveillance; responding to injury, and orchestrating immune responses (Amann et al., 2023). Key types include microglia, which are the resident immune cells of the brain, as well as macrophages and dendritic cells. Microglial originate from yolk sac progenitors during early development (Askew and Gomez-Nicola, 2018), while macrophages and dendritic cells can develop from bone marrow (Abdi et al., 2020; Cortez-Retamozo et al., 2012) or from the circulating monocytes (Sreejit et al., 2020; Marzaioli et al., 2020) during inflammation. A deeper understanding of these diverse cells and their origins is important for studying CNS health and disease.

Currently, the reviews on conventional (CD4^+^ and CD8^+^) and invariant (NKT and MAIT) T cells in neurological diseases (Wyatt-Johnson et al., 2024; Wyatt-Johnson and Brutkiewicz, 2020; DeMaio et al., 2022; Evans et al., 2019) focus specifically on T cells and tend to overlook the importance of the antigen-presenting molecules themselves. Here, we review the scientific literature in terms of studies where microglial MHC or MHC-like molecules were investigated in a variety of CNS diseases (also summarized in Table 1). As we discovered, such studies are not very numerous. Thus, we see many opportunities to include the analysis of antigen presenting molecules in several neurological disorders, which can advance the possible identification of novel therapeutic targets for these various diseases.

**Table 1:** Classical and non-classical MHC molecules in neurodegenerative diseases and disorders.

**Neurological disease/disorder**	**MHC I**	**MHC II**	**MR1**	**CD1d**
Alzheimer's disease and related dementia	In both AD and aging, there is an observed increase in the expression of microglial MHC class I molecules in human and mouse models (Kellogg et al., 2023; Zhou et al., 2020)	Increased MHC II expression around dense-core plaques in post-mortem human tissue (Hendrickx et al., 2017); MHC II-deficient mice demonstrated worsening AD pathology (Mittal et al., 2019)	Microglia MR1 expression increased on microglia closer to amyloid plaques in both mice and human models (Wyatt-Johnson et al., 2023)	CD1d neutralization did not impact the cognitive function of in mouse models of AD, but did reduce neuroinflammation in LBD mouse models (Iba et al., 2024)
Amyotrophic lateral sclerosis	Dual role for MHC I in ALS; can either have a beneficial effect (Nardo et al., 2018; Oliveira et al., 2004; Tomiyama et al., 2023; Song et al., 2016) or a pathogenic contribution (Nardo et al., 2018)	Increased HLA genes in the Glia subtype of ALS (Eshima et al., 2023)	Not reviewed here	Not reviewed here
Huntington's disease	Not reviewed here	Post-mortem HD brain sections showed MHC II present on activated microglia (Sapp et al., 2001)	Not reviewed here	Not reviewed here
Multiple sclerosis	Mediate a protective effect (Bergamaschi et al., 2010; Friese et al., 2008)	MHC II (HLA-DR) expression was found on CD68^+^ microglia near lesion areas (Hendrickx et al., 2017; Luchetti et al., 2018; Bo et al., 1994)	Increased expression in associated lesions of the brain and disease progression (Salou et al., 2016; Wyatt-Johnson et al., 2024; Dedoni et al., 2023)	Present in areas of active demyelination and along the borders of active lesions (Muir et al., 2020)
Parkinson's disease	Research on MHC class I expression in microglia remains particularly limited, while evidence indicates that neuronal MHC I levels are elevated in PD (Wang et al., 2021)	MHC II-deficient mice injected with human α-synuclein had reduced dopaminergic neuron loss (Harms et al., 2013) and in rats injected with human α-synuclein there was co-localization of MHC II with α-synuclein (Jimenez-Ferrer et al., 2021)	Not reviewed here	Not reviewed here

## MHC class I

MHC class I (MHC I) molecules play a central role in the immune system, as they present foreign peptides to T cells (Wieczorek et al., 2017). MHC class I molecules are expressed on all nucleated cells. They present endogenously-synthesized antigens from viruses or other pathogens that have been degraded into peptide-sized fragments by the proteasome in the cytosol and transported to the cell surface (Hewitt, 2003; Pearce et al., 2004). These eight or nine amino acid peptides are recognized by CD8^+^ T cells, which are cytotoxic in nature and eliminate infected APCs, thereby protecting the host (Rock et al., 2016).

In the normal human brain, CD8^+^ T cells are present at relatively low levels, primarily localizing in the white matter, perivascular spaces, and brain parenchyma (Smolders et al., 2013; Moreno-Valladares et al., 2020). However, in various neurodegenerative disorders, evidence shows that these cells are found at much higher levels, suggesting significant infiltration and active involvement in disease pathology. This shift of CD8^+^ T cell levels indicates a potential role in mediating immune responses within the CNS, which can be both protective and detrimental, depending on the context of the disease (Mensurado et al., 2023; Unger et al., 2020; Kimura et al., 2024).

MHC class I molecules are heterodimeric proteins composed of a peptide-binding heavy chain (α chain)—which forms the peptide-binding site central for antigen presentation—and a light chain known as β2-microglobulin—which stabilizes the antigen/T cell interaction (Hewitt, 2003; Muntjewerff et al., 2020). In this part of the review, we will explore the role of MHC class I molecules on microglia/macrophages and their interactions with CD8^+^ T cells across various neurodegenerative disorders.

### Alzheimer's disease and related dementias (ADRD)

In Alzheimer's disease (AD), the elevated expression of MHC class I molecules calls for further investigation to understand how these immune responses influence neurodegeneration. AD is the most prevalent form of dementia, primarily defined by the pathological accumulation of two aberrant proteins: amyloid-beta (Aβ) and hyperphosphorylated tau, together with neuroinflammation (O'Brien and Wong, 2011). Multiple lines of evidence suggest that increased levels of microglial MHC I molecules in AD may play a critical role in disease progression (Kellogg et al., 2023; Kim et al., 2023; Tooyama et al., 1990). Indeed, in the brains of AD patients, MHC I expression has been detected in microglia, albeit at a relatively low level (Tooyama et al., 1990). More recently, it has been demonstrated that microglial MHC I is expressed at significantly higher levels compared to other CNS cell types and that this expression increases with aging in both human and mouse models (Kellogg et al., 2023). Moreover, in the 5XFAD mouse model of AD, Zhou et al. (2020) found a significant upregulation of MHC I genes compared to wild-type (WT) mice, emphasizing their relevance in AD pathology. Furthermore, Goddery et al. (2021) determined that MHC I-expressing microglia are primary drivers of CD8^+^ immune cell infiltration and responses within the brain. Consistent with this, several studies have reported increased numbers of parenchymal and peripheral CD8^+^ T cells in AD patients, shedding light on a potential role for MHC class I molecules in AD (Larbi et al., 2009; Lueg et al., 2015; Unger et al., 2020). Additionally, there is evidence that neurons express MHC class I molecules under certain pathological conditions, including AD, and that amyloid beta plays a role in regulating the expression of MHC-I molecules (Kim et al., 2023). Although less is known about tau protein as an antigen in the context of CD8^+^ T cell recognition, it might also undergo processing and presentation in a similar manner, especially in stressed neurons. Evidence from one study indicates that reducing MHC-I expressions alleviates tau pathology in primary neurons expressing ApoE4 and in the hippocampi of mice with tau pathology (Zalocusky et al., 2021). Another study found that activated microglia in tauopathy show a notable increase in MHC-I expression (Chen et al., 2023). Together, these findings suggest that investigating MHC I-mediated immune responses in microglia and neurons could provide valuable insights into AD progression and potential therapeutic targets.

### Amyotrophic lateral sclerosis

The expression of MHC class I molecules in ALS suggests a multifaceted role that calls for additional investigation to untangle its diverse effects on disease pathology. Amyotrophic lateral sclerosis (ALS) is a fatal, progressive neurological disorder that targets both upper and lower motor neurons. Microglia have been found to have a pivotal role in modulating the neuroinflammatory process in ALS (Vahsen et al., 2023; Barreto-Núñez et al., 2024). Notably, work by Nardo et al. (2018), using β2-microglobulin-deficient SOD1G^93A^ mutant mice—obtained by crossing female C57BL6.129P2-B2mtm1Unc/J (B2M KO) mice with C57BL/6JSOD1^G93A^male mice— demonstrated that the lack of both spinal microglial MHC I and CD8^+^ T cell infiltration led to enhanced survival, delayed disease progression, and a significant reduction in the proinflammatory response. In contrast, in the sciatic nerves of these mice, the outcomes were the opposite, suggesting a complex role for these immune components in ALS pathology (Nardo et al., 2018). Consistent with this, microglia near motor neurons showed an increased expression level of MHC I in C57SOD1G^93A^ mice during disease progression (Nardo et al., 2013; Chiarotto et al., 2017). On the other hand, it has also been reported that neuronal MHC I expression has a beneficial effect on axonal regeneration and neuron survival overall (Oliveira et al., 2004; Tomiyama et al., 2023) and can protect against astrocyte-mediated neurotoxicity (Song et al., 2016). Overall, these findings suggest a dual role for MHC I in ALS, which may vary depending upon its site-specific expression and stage of disease.

### Multiple sclerosis

Studies on multiple sclerosis (MS) underscore the influence of MHC molecules, with emerging evidence suggesting a protective role for certain MHC class I alleles, though the mechanisms remain unclear. MS is a progressive autoimmune neurodegenerative disease that is typically associated with demyelination and the infiltration of immune cells into the CNS. Given the inflammatory profile of MS, numerous studies have highlighted the importance of MHC molecules in MS pathology (Ramagopalan and Ebers, 2009; Maghbooli et al., 2020). However, the precise mechanism(s) by which different classes of MHC molecules can significantly alter MS pathology require further attention. Whereas more attention has been focused on the role of MHC II molecules in MS, which will be discussed in detail later in this review, a few studies have highlighted the role of MHC I in MS disease pathology. Indeed, work by Bergamaschi et al., found that, in an Italian cohort of MS patients and controls, a specific (and common) HLA-class I allele (HLA-A^*^02) mediated a protective effect, thus reducing MS risk in this population (Bergamaschi et al., 2010). Similarly, another study also demonstrated a similar protective potential for the same HLA class I allele in regulating the risk of MS in a humanized mouse model of MS (Friese et al., 2008). Given the current knowledge of the impact of HLA class I alleles on MS susceptibility and pathology, further research is still needed to examine how MHC I molecules on cells like microglia may alter MS pathology. This will help us better understand the genetic basis of MS susceptibility and identify potential avenues for future studies and clinical applications.

### Parkinson's disease

The involvement of MHC class I molecules in Parkinson's disease (PD) has gained attention, with increased expression seen in neurons, but more research is needed to explore their impact on microglial-mediated inflammation. PD is another prevalent progressive neurodegenerative disease that predominantly targets the dopaminergic neurons, thus impairing motor functions. Although neuroinflammation is known to significantly contribute to PD pathology, the underlying mechanisms regulating these inflammatory responses are not yet fully understood. Interestingly, it was previously described that the expression levels of neuronal MHC class I were increased in PD (Wang et al., 2021). However, research into the role of MHC class I molecules on microglia in PD is particularly limited. Thus, addressing this knowledge gap will be critical for a more comprehensive understanding of the disease.

## MHC class II

MHC class II (MHC II) molecules present antigens to CD4^+^ T cells. MHC II is a cell surface glycoprotein with two heavy chains, each with two domains that are encoded by three different human leukocyte antigen (HLA) subclasses -DP, -DQ, and -DR (Buxade et al., 2018; van Lith et al., 2010). In the endoplasmic reticulum, the invariant chain (Ii) associates with MHC II molecules, with part of Ii, called CLIP, binding to the antigen-binding groove. This protects the MHC II molecules from binding peptides before they can interact with the correct antigenic peptides. Upon intracellular transport to late endosomal compartments, CLIP is exchanged with an antigenic peptide by HLA-DM (human) or H2-M (mouse) (Roche and Furuta, 2015). The >20 amino acid peptides that MHC II molecules present are from exogenous sources (i.e., outside of the cell) (Buxade et al., 2018). These peptides are then presented to CD4^+^ T cells, which are found in the brain even under healthy “non-disease” conditions (Pasciuto et al., 2020). The following sections will discuss how MHC II molecules on microglia or macrophages contribute to neurodegenerative diseases.

### Alzheimer's disease and related dementias

In AD, the genes encoding the HLA-DRB1 and HLA-DRB5 MHC II molecules are considered to be AD risk genes (Patel et al., 2021; Mathys et al., 2019) and overall MHC II expression is found to be elevated in ADRD pathology. In histological analyses of AD patient vs. non-AD brains, MHC II molecule protein expression is elevated on microglia that positively stain with the lectin *Ricinus communis* agglutinin—particularly microglia clustered around senile plaques and neurofibrillary tangles (Perlmutter et al., 1992). Similarly, in another cohort of post-mortem AD brain tissue, there was an increase in HLA-DR staining around dense-core plaques, but this did not co-localize with IBA1 or CD68 reactive (Hendrickx et al., 2017), however, this could be due to the expression pattern of these molecules and the difficulty of staining human tissue. Using RNA sequencing analyses, “disease-associated microglia” (DAMs) have been found in AD and shown to have increased expression of MHC II (Keren-Shaul et al., 2017; Mathys et al., 2019). Interestingly, in CK-p25 mice which have many hallmark features of AD, there are two types of DAMs which are defined by increased expression of MHC II molecules and Type 1 IFNs, which can be produced by macrophages (Mathys et al., 2019). In the 5XFAD mouse model of AD, the injection of cultured Aβ-specific CD4^+^ T helper type 1 cells directly into the brain, resulted in an increase in the colocalization of IBA1^+^MHC II^+^ cells with Aβ (Mittal et al., 2019). These cells also had a much more amoeboid morphology rather than ramified and the MHC II^+^ cells had a higher co-localization to Aβ compared to MHC II- cells (Mittal et al., 2019). Interestingly, 5XFAD mice crossed with MHC II-deficient mice had an increase in the IL-6 and IFN-γ proinflammatory cytokines at 6 months of age, along with an increased plaque load (Mittal et al., 2019). These data indicate that the antigen-presenting molecules are crucial in AD pathology by potentially triggering the clearance of Aβ. Moreover, 5XFAD mice treated with a flavonoid from *Hibiscus sabdariffa*, Gossypetin, had a reduction in both Aβ and IBA1^+^MHC II^+^ cells (Jo et al., 2022). In the tauopathy mouse model TE4, treatment with PLX3397 reduced IBA1^+^ cells and MHC II^+^ cells and reduced CD3^+^ T cells. Treatment with anti-CD4 and anti-CD8 antibodies in TE4 mice also reduced MHC II^+^ cells in the dentate gyrus (Chen et al., 2023). In two transgenic rat lines expressing human truncated tau protein, the spontaneously hypertensive rat only had a small percentage of microglia expressing MHC II (1.6%), while the Wistar-Kyoto background has almost a fourth of microglia expressing MHC II (23.2%) (Stozicka et al., 2010). Interestingly, the spontaneously hypertensive rat had more overall neurofibrillary load (Stozicka et al., 2010). More recently, in APOE3 and APOE4 mouse models transfected with humanized tau, only the APOE4 mice showed an increased surface level of MHC II on CD11b^+^ CD45^−^ microglia (Lu et al., 2024). Furthermore, in these mice, there was a connection between APOE4 enhancing cholesterol accumulation in microglia that have elevated levels of MHC II, and in microglia where capsaicin was used to increase calcium levels in the endoplasmic reticulum, they had reduced MHC II expression (Lu et al., 2024). Overall, these studies suggest that MHC II molecules may play a role in altering the Aβ plaque load and tau in ADRD.

### Amyotrophic lateral sclerosis

In ALS gene expression changes for MHC II have shown to be increased in certain types of ALS. ALS is associated with the RNA/DNA-binding proteins, FUS, EWSR1, TAF15, and MATR3. These were found to influence MHC II-mediated antigen presentation via regulation of the MHC class II transactivator (CIITA) (Chi et al., 2023). In a subtype of ALS called Glia, there is a significant increase in *HLA-DOA* gene expression in comparison to controls, frontotemporal dementia, and the two other subtypes of ALS OX (oxidative stress dysregulation) and TD (dysregulation of transcription) (Eshima et al., 2023). This is particularly important as HLA-DOA forms a heterodimer with HLA-DOB in lysosomes which helps mediate peptide loading onto MHC II molecules. Moreover, in ALS-Glia, there is a high contribution of glial cells, where the “DAM” microglia found in AD are also observed here (Eshima et al., 2023). Although there has been little work on the role of MHC II molecules in ALS, existing data suggest a role for MHC class II molecules in disease progression and as a potential therapeutic target.

### Huntington's disease

Huntington's disease (HD) is an inherited disorder that causes the progressive loss of neurons with very little analysis of MHC II, only showing histological increases of MHC II. Few studies have analyzed MHC II-encoding or related genes in HD. In higher grade HD patient post-mortem brain sections, who were not diagnosed with any other neurological disease, activated microglia are MHC II^+^ (Sapp et al., 2001). This is in contrast to the number of peripheral MHC II^+^ macrophages which remained unchanged in HD patients (Pido-Lopez et al., 2018; Trager et al., 2014). These studies therefore indicate possible functional effects of MHC II molecule expression in microglia in HD.

### Multiple sclerosis

In MS the MHC II HLA genes were overall found to be increased. MS has some alleles in the *HLA-DQB1, HLA-DQA1*, and *HLA-DRB1* loci are associated with an increased risk of developing MS. Interestingly, the *HLA-DRB1* haplotype in MS patients is different, depending upon the geographical region in which they reside (Ramagopalan and Ebers, 2009). Additionally, the *HLA-DR15* allele has one of the strongest risk associations for MS (Martin et al., 2021). Commonly, in post-mortem MS tissue, HLA expression is used as a diagnostic for inflammatory activation in lesion areas (Luchetti et al., 2018). In post-mortem MS brain sections, HLA-DR-DQ-DP was found most predominately in white matter areas, although it was still present in the gray matter (Hendrickx et al., 2017). Of note, HLA-DR-DQ-DP staining was found particularly more in active MS lesions compared to inactive MS lesions across different cohorts (Luchetti et al., 2018; Hendrickx et al., 2017; Bo et al., 1994). This HLA-DR-DQ-DP staining was co-localized frequently with CD68, a lysosomal marker used to help measure activation, but less with IBA1^+^ cells, which were amoeboid in shape, but did co-localize with IBA1^+^ foamy macrophages (Hendrickx et al., 2017; Bo et al., 1994). Although this staining did not co-localize frequently with IBA1^+^ microglia/macrophages, it did not co-localize at all with astrocytic or endothelial markers (Bo et al., 1994). Interestingly, the microglia clusters (or nodules) in MS express more HLA-DR-DP-DQ than in post-mortem human stroke microglia clusters (van den Bosch et al., 2024). These clusters in MS are also shown to contain partially demyelinated axons (van den Bosch et al., 2024). These studies demonstrate the importance of understanding microglia/macrophages near lesion areas, as well as the importance of which markers are used to assess the lesion areas. Overall, various MHC II alleles are associated with a higher incidence of MS, the microglia/macrophages with the highest expression of these alleles are located near lesion areas. Understanding microglia/macrophages in lesion areas and their expression of MHC II could potentially lead to better therapeutic targets, more work and advancement of technology is needed to understand how these cells may initiate or contribute to MS lesions.

### Parkinson's disease

In PD, MHC II levels are increased in animal models. These increased levels of MHC II molecules have been observed in mouse models of PD using unilateral injections of full length human α-synuclein into the substantia nigra pars compacta (Harms et al., 2013). Injection of human α-synuclein into MHC II-deficient mice prevented this increase as well as the activation of CD11b^+^ microglia; dopaminergic neuron loss was also reduced (Harms et al., 2013). In Dark agouti (DA) and DA.VRA4-congenic rats, the latter of which display lower levels of the MHC II regulatory gene *Mhc2ta* (the rat equivalent of CTIIA), were similarly injected with human α-synuclein. As expected, compared to DA rats, there was reduced expression of MHC II molecules in the DA.VRA4 rats. Interestingly, unlike the MHC II-deficient mice described above, there was more propagation of α-synuclein in the brains of DA.VRA4 rats (Jimenez-Ferrer et al., 2021). Notably, in the areas of α-synuclein propagation, there were more IBA1^+^MHC II^+^ vs. IBA1^+^MHC II- cells. Additionally, the IBA1^+^MHC II^+^ cells had a more amoeboid morphology (Jimenez-Ferrer et al., 2021). DA.VRA4 rats also showed the greatest cell loss in the nigra and developed a progressive forelimb akinesia (Jimenez-Ferrer et al., 2021). Regardless of rat type, MHC II^+^ cells colocalized with α-synuclein (Jimenez-Ferrer et al., 2021). When nude rats, which lack T cells, were injected with human α-synuclein, there was no increase in MHCII (Subbarayan et al., 2020). Of note, the lower levels of *Mhc2ta* (i.e., CIITA) expression in DA.VRA4 rats are correlated with higher levels of TNF (Fredlund et al., 2024). This is a bit counter-intuitive, as one would expect CIITA (and thereby, MHC II) and TNF to be regulated in parallel during an inflammatory response. Overall, observations demonstrating that α-synuclein propagation and its impact on glial activation, as well as neuronal loss, may be influenced by MHC class II molecules.

## MR1

The MHC I like molecule MR1 is a non-polymorphic β2-microglobulin-dependent class Ib molecule expressed both intracellularly and on the surface of several types of cells. MR1 is required for the development, expansion and activation of T cells with an invariant T cell receptor α chain (Vα 7.2/Jα 33 in humans and Vα19-Jα33 in mice), called mucosal-associated invariant T (MAIT) cells, which have been found in the brain in human neurological diseases (Shrinivasan et al., 2024; Wyatt-Johnson et al., 2024; Treiner et al., 2003). In terms of antigen presentation, MR1 covalently binds intermediates derived from the microbial metabolism of vitamin B intracellularly and then presents them on the cell surface to MAIT cells (Lamichhane and Ussher, 2017). It is important to note that MR1 is highly conserved in mammals, though expression levels, ligand loading and trafficking pathways have shown some variation between cell lines and host species (Lamichhane and Ussher, 2017). Whereas roles for MR1 in infections and autoimmune responses have been established, considerably less information is available about MR1 in the context of neurodegenerative diseases in which neuroinflammation is often a characteristic. For example, chronic MR1-mediated MAIT cell activation and the subsequent secretion of proinflammatory cytokines have been shown to worsen the outcome of some neurodegenerative diseases (Landry and Embers, 2022). Below, we discuss what is known about the contributions of MR1 to various neurodegenerative disorders.

### Alzheimer's disease and related dementias

Determining the role of MR1, if any, in homeostatic or neuroinflammatory processes is ongoing. The low permeability of the blood-brain barrier (BBB) is essential for maintaining brain homeostasis (Huang et al., 2020). The breakdown of the BBB into a “leakier” state is an early-stage event of the disease process in Alzheimer's disease (Wyatt-Johnson and Brutkiewicz, 2020). Otherwise, toxins, cytokines and immune cells could infiltrate into the brain and possibly trigger excessive microglial activation, resulting in neuroinflammation (Zhang et al., 2022). It has been shown that MR1 has a role in the stability of the BBB of C57BL/6 mice as they age. For example, one study demonstrated that MR1-deficient mice (lacking both MR1 and MAIT cells) have an increase in ROS accumulation and exhibit meningeal barrier leakage compared to WT mice. These results imply that MR1 is a protective factor for maintaining the meningeal barrier structure in WT mice (Zhang et al., 2022). Further experiments showed that IBA^+^ cells were increased in the hippocampus and cortex of mice lacking MR1 (Zhang et al., 2022). Excessive microglial activation may contribute to neuroinflammation through the release of an array of proinflammatory cytokines like IL-6, IL-1β, IL-18, TNF-α, and IFN-γ, all capable of causing injury to neurons (Landry and Embers, 2022). In fact, reduced cognitive function was observed in MR1 KO mice in both Y-Maze and Morris Water Maze assessments. This reduction was ameliorated after the transfer of MAIT cells to MR1 KO mice (Zhang et al., 2022); thus, these data suggest that MR1-independent, MAIT cell-dependent responses can have an impact in the CNS. In the 5XFAD mouse model of AD, MR1-deficient 5XFAD mice showed a significantly delayed accumulation of Aβ in the hippocampus until 8 months of age, when compared to 5XFAD mice that are MR1^+^. In the cortex, this significant difference in the Aβ burden between 5XFAD and MR1-deficient 5XFAD mice was still present at 8 months (Wyatt-Johnson et al., 2023). Moreover, higher levels of MR1 were found in IBA1^+^ microglia/macrophages that were closer to amyloid plaques than those at greater distances or as compared to WT mouse IBA1^+^ cells. Notably, this result was also demonstrated in the brain tissue of AD patients as compared to non-AD controls (Wyatt-Johnson et al., 2023). Considering that the gut microbiome is altered in AD patients (Bello-Corral et al., 2023) and the importance of MAIT cells in maintaining gut immune homeostasis (Jabeen and Hinks, 2023), examined together, these data suggest that MR1 has a possible role in AD pathology.

### Multiple sclerosis

Expression of MR1 is increased in the brain lesions of Multiple Sclerosis patients. Like most of the neurodegenerative diseases discussed so far, there is a limited amount of data discussing any correlation between MR1 expression and the progression or overall pathology of MS. Much of what has been published is in relation to MAIT cell activation or the overall MAIT cell population, with little analysis on MR1 itself (Lv et al., 2023). There is an increased expression of MR1 localized in MS-associated brain lesions regardless of the form of MS (i.e., Primary Progressive, Progressive Relapsing, Relapsing-Remitting, and Secondary Progressive) in which the patient presents. Enhanced MR1 expression was detected in patient tissue associated with the three states of MS white matter lesions: (1) active, (2) chronic active, and (3) normal but notable for inflammatory mediators (Salou et al., 2016). It was found that with the increase in MR1 expression, the number of MAIT cells in the CSF increased in those with MS. It is understandable, considering the proinflammatory nature of the MR1/MAIT cell axis, that the increased expression of MR1 has often been correlated with a worsening prognosis (Salou et al., 2016). Thus, overall, MR1 levels are positively correlated with lesion areas in the brains of human MS patients, suggesting possible contributions of this MHC class I-like molecule in MS pathology development and disease progression.

## CD1d

Like MR1, CD1d is a constitutively expressed MHC class I–like molecule with some non-classical functions capable of presenting antigens to innate T cells, such as invariant NKT (iNKT) cells. iNKT cells are CD1d-restricted T cells with an invariant α chain rearrangement (identified as Vα14-Jα18 in mice and Vα24-Jα18 in humans) with the ability to produce both pro- and anti-inflammatory cytokines, and similar to MAIT cells have been found in the brain in neurological diseases (Wyatt-Johnson et al., 2024). Unlike MR1, CD1d presents glycolipids and phospholipids; these include such lipids as α-galactosylceramide (α-GalCer), its derivative OCH (α-GalCer with a truncated sphingosine chain) (Miyamoto et al., 2001), or the myelin sheath component sulfatide (Brutkiewicz et al., 2018; Treiner et al., 2003; Wyatt-Johnson et al., 2024). Before CD1d can present activating lipid antigens it must be synthesized in the ER and loaded with non-reactive antigens before trafficking through the Golgi and ultimately reaching the surface of APCs (Brutkiewicz et al., 2018; Iba et al., 2024). When needed, CD1d recycles back into the cell where the non-reactive lipid is exchanged with NKT cell-activating lipid antigens. As CD1d is required for the selection of NKT cells, the use of CD1d KO and Jα18 KO/Traj18 KO (only lacking NKT cells, but are CD1d^+^) mouse models act as valuable tools for investigating possible roles in disease (Cui and Wan, 2019; Chandra et al., 2015). CD1d and the subsequent activation of iNKT cells have been studied in many infections, autoimmune disorders and cancers (Brutkiewicz et al., 2018). That said, there are limited investigations into their role in neurodegenerative disorders, with only a handful of studies focusing on CD1d changes in neurodegeneration (Iba et al., 2024; Muir et al., 2020; Parekh et al., 2013); these include using an anti-CD1d mAb to reduce the number of NKT cells in α-synuclein transgenic mice and elevated astrocyte CD1d expression in MS.

### Alzheimer's disease and related dementias

The response to antibody-mediated blocking of CD1d varies between different dementia mouse model systems. In Alzheimer's disease, there have been no studies focused on changes in CD1d expression in the brains of patients or how their levels may compare to healthy controls (Sieberts et al., 2020). There has been at least one study utilizing the 3xTg-AD mouse model that indicated CD1d-neutralizing antibodies did not significantly affect the cognitive function of mice (Iba et al., 2024). These studies therefore imply that CD1d does not contribute to AD, although a simple explanation could be that the anti-CD1d antibodies used do not block NKT cell/CD1d interactions. In contrast, an interesting study of another form of dementia, Lewy Body Disease (LBD) in α-synuclein transgenic mice (a model for LBD), showed that treatment with anti-CD1d antibodies decreased the numbers of NKT cells and reduced neuroinflammation (Iba et al., 2024). Thus, the disparate responses to CD1d blocking in AD vs. LBD model systems emphasize the need for further research into the effects of targeting CD1d in multiple forms of dementia.

### Multiple sclerosis

The role of CD1d in MS is not well understood. In a study of MS patient tissue, immunoreactive CD1d was largely found in areas of active CNS demyelination compared to controls (Muir et al., 2020). Moreover, the percentage of CD1d-positive cells was higher in active lesions. It was also noted that CD1d-positive cell density was greatest at the edges of lesions as compared to the center (Muir et al., 2020). Overall, in MS CD1d appears to be associated with lesions but the how it may impact these areas remains unknown.

## Conclusion

The functional expression of MHC and MHC-like molecules has biological consequences in terms of host defense and also, tissue damage because of bystander killing by cytotoxic effector cells or the induction of proinflammatory cytokine production, which can be especially significant in the CNS. That being said, our understanding of these connections within CNS disorders is still limited. It seems that the more we know about the contributions of these antigen presenting molecules in CNS diseases, such studies may reveal strategies to mitigate the overexpression of MHC and MHC-like molecules, potentially suppressing neuroinflammation and its consequent effects on pathology. Such investigations have the exciting potential to lead to novel MHC/MHC-like molecule-based therapeutic approaches in various CNS disorders.

## References

[B1] Abdi K. Thomas L. M. Laky K. Abshari M. Matzinger P. Long E. O.. (2020). Bone marrow–derived dendritic cell cultures from RAG–/– mice include IFN-γ-producing NK cells. ImmunoHorizons 4, 415–419. 10.4049/immunohorizons.2000011 32665300 PMC7454002

[B2] Amann L. Masuda T. Prinz M. (2023). Mechanisms of myeloid cell entry to the healthy and diseased central nervous system. Nat. Immunol. 24, 393–407. 10.1038/s41590-022-01415-8 36759712

[B3] Askew K. Gomez-Nicola D. (2018). A story of birth and death: Insights into the formation and dynamics of the microglial population. Brain Behav. Immun. 69, 9–17. 10.1016/j.bbi.2017.03.009 28341583

[B4] Barreto-Núñez R. Béland L. C. Boutej H. Picher-Martel V. Dupré N. Barbeito L.. (2024). Chronically activated microglia in ALS gradually lose their immune functions and develop unconventional proteome. Glia 72, 1319–1339. 10.1002/glia.24531 38577970

[B5] Bello-Corral L. Alves-Gomes L. Fernández-Fernández J. A. Fernández-García D. Casado-Verdejo I. Sánchez-Valdeón L.. (2023). Implications of gut and oral microbiota in neuroinflammatory responses in Alzheimer's disease. Life Sci. 333:122132. 10.1016/j.lfs.2023.122132 37793482

[B6] Bendelac A. Savage P. B. Teyton L. (2007). The biology of NKT cells. Annu. Rev. Immunol. 25, 297–336. 10.1146/annurev.immunol.25.022106.141711 17150027

[B7] Bergamaschi L. Leone M. A. Fasano M. E. Guerini F. R. Ferrante D. Bolognesi E.. (2010). HLA-class I markers and multiple sclerosis susceptibility in the Italian population. Genes Immun. 11, 173–80. 10.1038/gene.2009.101 19907433 PMC2834350

[B8] Birkinshaw R. W. Kjer-Nielsen L. Eckle S. B. Mccluskey J. Rossjohn J. (2014). MAITs, MR1 and vitamin B metabolites. Curr. Opin. Immunol. 26, 7–13. 10.1016/j.coi.2013.09.007 24556396

[B9] Bo L. Mork S. Kong P. A. Nyland H. Pardo C. A. Trapp B. D.. (1994). Detection of MHC class II-antigens on macrophages and microglia, but not on astrocytes and endothelia in active multiple sclerosis lesions. J. Neuroimmunol. 51, 135–146. 10.1016/0165-5728(94)90075-2 8182113

[B10] Brutkiewicz R. R. (2006). CD1d ligands: the good, the bad, and the ugly. J. Immunol. 177, 769–775. 10.4049/jimmunol.177.2.769 16818729

[B11] Brutkiewicz R. R. Yunes-Medina L. Liu J. (2018). Immune evasion of the CD1d/NKT cell axis. Curr. Opin. Immunol. 52, 87–92. 10.1016/j.coi.2018.04.021 29734045 PMC6004260

[B12] Buxade M. Huerga Encabo H. Riera-Borrull M. Quintana-Gallardo L. Lopez-Cotarelo P. Tellechea M.. (2018). Macrophage-specific MHCII expression is regulated by a remote Ciita enhancer controlled by NFAT5. J. Exp. Med. 215, 2901–2918. 10.1084/jem.20180314 30327417 PMC6219740

[B13] Chandra S. Zhao M. Budelsky A. De Mingo Pulido A. Day J. Fu Z.. (2015). A new mouse strain for the analysis of invariant NKT cell function. Nat. Immunol. 16, 799–800. 10.1038/ni.3203 26075912 PMC4711267

[B14] Chen X. Firulyova M. Manis M. Herz J. Smirnov I. Aladyeva E.. (2023). Microglia-mediated T cell infiltration drives neurodegeneration in tauopathy. Nature 615, 668–677. 10.1038/s41586-023-05788-0 36890231 PMC10258627

[B15] Chi B. Ozturk M. M. Paraggio C. L. Leonard C. E. Sanita M. E. Dastpak M.. (2023). Causal ALS genes impact the MHC class II antigen presentation pathway. Proc. Natl. Acad. Sci. USA. 120:e2305756120. 10.1073/pnas.2305756120 37722062 PMC10523463

[B16] Chiarotto G. B. Nardo G. Trolese M. C. França M. C. Jr. Bendotti C. Rodrigues De Oliveira A. L. (2017). The emerging role of the major histocompatibility complex class I in amyotrophic lateral sclerosis. Int. J. Mol. Sci. 18:2298. 10.3390/ijms18112298 29104236 PMC5713268

[B17] Cortez-Retamozo V. Etzrodt M. Pittet M. J. (2012). Regulation of macrophage and dendritic cell responses by their lineage precursors. J. Innate Immun. 4, 411–423. 10.1159/000335733 22433183 PMC3498093

[B18] Cui Y. Wan Q. (2019). NKT cells in neurological diseases. Front. Cell. Neurosci. 13:245. 10.3389/fncel.2019.00245 31231193 PMC6558384

[B19] Dausset J. Contu L. (1980). Is the MHC a general self-recognition system playing a major unifying role in an organism? Hum. Immunol. 1, 5–17. 10.1016/0198-8859(80)90004-X 6455393

[B20] Dedoni S. Scherma M. Camoglio C. Siddi C. Dazzi L. Puliga R.. (2023). An overall view of the most common experimental models for multiple sclerosis. Neurobiol Dis. 184:106230. 10.1016/j.nbd.2023.106230 37453561

[B21] DeMaio A. Mehrotra S. Sambamurti K. Husain S. (2022). The role of the adaptive immune system and T cell dysfunction in neurodegenerative diseases. J. Neuroinflammation 19:251. 10.1186/s12974-022-02605-9 36209107 PMC9548183

[B22] Eshima J. O'connor S. A. Marschall E. Consortium N. A. Bowser R. Plaisier C. L.. (2023). Molecular subtypes of ALS are associated with differences in patient prognosis. Nat. Commun. 14:95. 10.1038/s41467-022-35494-w 36609402 PMC9822908

[B23] Evans F. L. Dittmer M. De La Fuente A. G. Fitzgerald D. C. (2019). Protective and regenerative roles of T cells in central nervous system disorders. Front. Immunol. 10:2171. 10.3389/fimmu.2019.02171 31572381 PMC6751344

[B24] Fabry Z. Raine C. S. Hart M. N. (1994). Nervous tissue as an immune compartment: the dialect of the immune response in the CNS. Immunol. Today 15, 218–224. 10.1016/0167-5699(94)90247-X 8024682

[B25] Fredlund F. Jimenez-Ferrer I. Grabert K. Belfiori L. F. Luk K. Swanberg M.. (2024). Ciita regulates local and systemic immune responses in a combined rAAV-alpha-synuclein and preformed fibril-induced rat model for Parkinson's disease. J. Parkinsons. Dis. 14, 693–711. 10.3233/JPD-240062 38728204 PMC11191526

[B26] Friese M. A. Jakobsen K. B. Friis L. Etzensperger R. Craner M. J. Mcmahon R. M.. (2008). Opposing effects of HLA class I molecules in tuning autoreactive CD8+ T cells in multiple sclerosis. Nat. Med. 14, 1227–1235. 10.1038/nm.1881 18953350

[B27] Frohman E. M. Van Den Noort S. Gupta S. (1989). Astrocytes and intracerebral immune responses. J. Clin. Immunol. 9, 1–9. 10.1007/BF00917121 2649507

[B28] Früh K. Yang Y. (1999). Antigen presentation by MHC class I and its regulation by interferon gamma. Curr. Opin. Immunol. 11, 76–81. 10.1016/S0952-7915(99)80014-4 10047537

[B29] Gelderblom M. Leypoldt F. Steinbach K. Behrens D. Choe C. U. Siler D. A.. (2009). Temporal and spatial dynamics of cerebral immune cell accumulation in stroke. Stroke 40, 1849–1857. 10.1161/STROKEAHA.108.534503 19265055

[B30] Giacomini P. Fisher P. B. Duigou G. J. Gambari R. Natali P. G. (1988). Regulation of class II MHC gene expression by interferons: insights into the mechanism of action of interferon (review). Anticancer Res. 8, 1153–61. 2464333

[B31] Goddery E. N. Fain C. E. Lipovsky C. G. Ayasoufi K. Yokanovich L. T. Malo C. S.. (2021). Microglia and perivascular macrophages act as antigen presenting cells to promote CD8 T cell infiltration of the brain. Front. Immunol. 12:726421. 10.3389/fimmu.2021.726421 34526998 PMC8435747

[B32] Godfrey D. I. Koay H. F. Mccluskey J. Gherardin N. A. (2019). The biology and functional importance of MAIT cells. Nat. Immunol. 20, 1110–1128. 10.1038/s41590-019-0444-8 31406380

[B33] Hanisch U. K. (2013). Proteins in microglial activation–inputs and outputs by subsets. Curr. Protein Pept. Sci. 14, 3–15. 10.2174/1389203711314010003 23441901

[B34] Harms A. S. Cao S. Rowse A. L. Thome A. D. Li X. Mangieri L. R.. (2013). MHCII is required for alpha-synuclein-induced activation of microglia, CD4 T cell proliferation, and dopaminergic neurodegeneration. J. Neurosci. 33, 9592–600. 10.1523/JNEUROSCI.5610-12.2013 23739956 PMC3903980

[B35] Hart M. N. Fabry Z. (1995). CNS antigen presentation. Trends Neurosci. 18, 475–481. 10.1016/0166-2236(95)92767-K 8592751

[B36] Healy L. M. Yaqubi M. Ludwin S. Antel J. P. (2020). Species differences in immune-mediated CNS tissue injury and repair: a (neuro)inflammatory topic. Glia 68, 811–829. 10.1002/glia.23746 31724770

[B37] Hendrickx D. A. E. Van Eden C. G. Schuurman K. G. Hamann J. Huitinga I. (2017). Staining of HLA-DR, Iba1 and CD68 in human microglia reveals partially overlapping expression depending on cellular morphology and pathology. J. Neuroimmunol. 309, 12–22. 10.1016/j.jneuroim.2017.04.007 28601280

[B38] Hewitt E. W. (2003). The MHC class I antigen presentation pathway: strategies for viral immune evasion. Immunology 110, 163–169. 10.1046/j.1365-2567.2003.01738.x 14511229 PMC1783040

[B39] Huang Z. Wong L. W. Su Y. Huang X. Wang N. Chen H.. (2020). Blood-brain barrier integrity in the pathogenesis of Alzheimer's disease. Front. Neuroendocrinol. 59:100857. 10.1016/j.yfrne.2020.100857 32781194

[B40] Iba M. Kwon S. Kim C. Szabo M. Horan-Portelance L. Lopez-Ocasio M.. (2024). Immunotherapy with an antibody against CD1d modulates neuroinflammation in an α-synuclein transgenic model of Lewy body like disease. J. Neuroinflammation 21:93. 10.1186/s12974-024-03087-7 38622654 PMC11017481

[B41] Jabeen M. F. Hinks T. S. C. (2023). MAIT cells and the microbiome. Front. Immunol. 14:1127588. 10.3389/fimmu.2023.1127588 36911683 PMC9995591

[B42] Jimenez-Ferrer I. Backstrom F. Duenas-Rey A. Jewett M. Boza-Serrano A. Luk K. C.. (2021). The MHC class II transactivator modulates seeded alpha-synuclein pathology and dopaminergic neurodegeneration in an in vivo rat model of Parkinson's disease. Brain Behav. Immun. 91, 369–382. 10.1016/j.bbi.2020.10.017 33223048

[B43] Jo K. W. Lee D. Cha D. G. Oh E. Choi Y. H. Kim S.. (2022). Gossypetin ameliorates 5xFAD spatial learning and memory through enhanced phagocytosis against Abeta. Alzheimers. Res. Ther. 14:158. 10.1186/s13195-022-01096-3 36271414 PMC9585741

[B44] Ju J. K. Cho Y. N. Park K. J. Kwak H. D. Jin H. M. Park S. Y.. (2020). Activation, deficiency, and reduced IFN-γ production of mucosal-associated invariant T cells in patients with inflammatory Bowel disease. J. Innate Immun. 12, 422–434. 10.1159/000507931 32535589 PMC7506267

[B45] Kaufman J. F. Auffray C. Korman A. J. Shackelford D. A. Strominger J. (1984). The class II molecules of the human and murine major histocompatibility complex. Cell 36, 1–13. 10.1016/0092-8674(84)90068-0 6198089

[B46] Keller A. N. Corbett A. J. Wubben J. M. Mccluskey J. Rossjohn J. (2017). MAIT cells and MR1-antigen recognition. Curr. Opin. Immunol. 46, 66–74. 10.1016/j.coi.2017.04.002 28494326

[B47] Kellogg C. M. Pham K. Machalinski A. H. Porter H. L. Blankenship H. E. Tooley K.. (2023). Microglial MHC-I induction with aging and Alzheimer's is conserved in mouse models and humans. bioRxiv. 10.1101/2023.03.07.531435 37393197 PMC10643718

[B48] Keren-Shaul H. Spinrad A. Weiner A. Matcovitch-Natan O. Dvir-Szternfeld R. Ulland T. K.. (2017). A unique microglia type associated with restricting development of Alzheimer's disease. Cell, 169, 1276–1290 e17. 10.1016/j.cell.2017.05.018 28602351

[B49] Kim M. S. Cho K. Cho M. H. Kim N. Y. Kim K. Kim D. H.. (2023). Neuronal MHC-I complex is destabilized by amyloid-β and its implications in Alzheimer's disease. Cell Biosci. 13:181. 10.1186/s13578-023-01132-1 37773139 PMC10540404

[B50] Kimura K. Nishigori R. Hamatani M. Sawamura M. Ashida S. Fujii C.. (2024). Resident memory-like CD8(+) T cells are involved in chronic inflammatory and neurodegenerative diseases in the CNS. Neurol. Neuroimmunol. Neuroinflamm. 11:e200172. 10.1212/NXI.0000000000200172 37949669 PMC10691221

[B51] Kjer-Nielsen L. Patel O. Corbett A. J. Le Nours J. Meehan B. Liu L.. (2012). MR1 presents microbial vitamin B metabolites to MAIT cells. Nature 491, 717–723. 10.1038/nature11605 23051753

[B52] Kreutzberg G. W. (1995). Microglia, the first line of defence in brain pathologies. Arzneimittelforschung 45, 357–360. 7763326

[B53] Lamichhane R. Ussher J. E. (2017). Expression and trafficking of MR1. Immunology 151, 270–279. 10.1111/imm.12744 28419492 PMC5461101

[B54] Landry R. L. Embers M. E. (2022). Does dementia have a microbial cause? NeuroSci. 3, 262–283. 10.3390/neurosci3020019 39483362 PMC11523730

[B55] Larbi A. Pawelec G. Witkowski J. M. Schipper H. M. Derhovanessian E. Goldeck D.. (2009). Dramatic shifts in circulating CD4 but not CD8 T cell subsets in mild Alzheimer's disease. J. Alzheimers. Dis. 17, 91–103. 10.3233/JAD-2009-1015 19494434

[B56] Lassmann H. Zimprich F. Rössler K. Vass K. (1991). Inflammation in the nervous system. Basic mechanisms and immunological concepts. Rev. Neurol. 147, 763–781. 1780606

[B57] Lehmann J. Härtig W. Seidel A. Füldner C. Hobohm C. Grosche J.. (2014). Inflammatory cell recruitment after experimental thromboembolic stroke in rats. Neuroscience 279, 139–54. 10.1016/j.neuroscience.2014.08.023 25168731

[B58] Lu J. Wu K. Sha X. Lin J. Chen H. Yu Z.. (2024). TRPV1 alleviates APOE4-dependent microglial antigen presentation and T cell infiltration in Alzheimer's disease. Transl. Neurodegener. 13:52. 10.1186/s40035-024-00445-6 39468688 PMC11520887

[B59] Luchetti S. Fransen N. L. Van Eden C. G. Ramaglia V. Mason M. Huitinga I.. (2018). Progressive multiple sclerosis patients show substantial lesion activity that correlates with clinical disease severity and sex: a retrospective autopsy cohort analysis. Acta Neuropathol. 135, 511–528. 10.1007/s00401-018-1818-y 29441412 PMC5978927

[B60] Lueg G. Gross C. C. Lohmann H. Johnen A. Kemmling A. Deppe M.. (2015). Clinical relevance of specific T-cell activation in the blood and cerebrospinal fluid of patients with mild Alzheimer's disease. Neurobiol. Aging 36, 81–89. 10.1016/j.neurobiolaging.2014.08.008 25277040

[B61] Lv M. Zhang Z. Cui Y. (2023). Unconventional T cells in brain homeostasis, injury and neurodegeneration. Front. Immunol. 14:1273459. 10.3389/fimmu.2023.1273459 37854609 PMC10579804

[B62] Maghbooli Z. Sahraian M. A. Naser Moghadasi A. (2020). Multiple sclerosis and human leukocyte antigen genotypes: focus on the Middle East and North Africa region. Mult. Scler. J. Exp. Transl. Clin. 6:2055217319881775. 10.1177/2055217319881775 31976083 PMC6956601

[B63] Martin R. Sospedra M. Eiermann T. Olsson T. (2021). Multiple sclerosis: doubling down on MHC. Trends Genet. 37, 784–797. 10.1016/j.tig.2021.04.012 34006391

[B64] Marzaioli V. Canavan M. Floudas A. Wade S. C. Low C. Veale D. J.. (2020). Monocyte-derived dendritic cell differentiation in inflammatory arthritis is regulated by the JAK/STAT Axis via NADPH oxidase regulation. Front. Immunol. 11:1406. 10.3389/fimmu.2020.01406 32733468 PMC7358435

[B65] Mathys H. Davila-Velderrain J. Peng Z. Gao F. Mohammadi S. Young J. Z.. (2019). Single-cell transcriptomic analysis of Alzheimer's disease. Nature 570, 332–337. 10.1038/s41586-019-1195-2 31042697 PMC6865822

[B66] Mensurado S. Blanco-Domínguez R. Silva-Santos B. (2023). The emerging roles of γδ T cells in cancer immunotherapy. Nat. Rev. Clin. Oncol. 20, 178–191. 10.1038/s41571-022-00722-1 36624304

[B67] Mittal K. Eremenko E. Berner O. Elyahu Y. Strominger I. Apelblat D.. (2019). CD4 T cells induce a subset of MHCII-expressing microglia that attenuates alzheimer pathology. iScience 16, 298–311. 10.1016/j.isci.2019.05.039 31203186 PMC6581663

[B68] Miyamoto K. Miyake S. Yamamura T. (2001). A synthetic glycolipid prevents autoimmune encephalomyelitis by inducing TH2 bias of natural killer T cells. Nature 413, 531–534. 10.1038/35097097 11586362

[B69] Moreno-Valladares M. Silva T. M. Garcés J. P. Saenz-Antoñanzas A. Moreno-Cugnon L. Álvarez-Satta M.. (2020). CD8(+) T cells are present at low levels in the white matter with physiological and pathological aging. Aging 12, 18928–18941. 10.18632/aging.104043 33049712 PMC7732290

[B70] Muir F. G. W. Samadi-Bahrami Z. Moore G. R. W. Quandt J. A. (2020). Expression of CD1d by astrocytes corresponds with relative activity in multiple sclerosis lesions. Brain Pathol. 30, 26–35. 10.1111/bpa.12733 31050367 PMC6916356

[B71] Muntjewerff E. M. Meesters L. D. Van Den Bogaart G. Revelo N. H. (2020). Reverse Signaling by MHC-I molecules in immune and non-immune cell types. Front. Immunol. 11:605958. 10.3389/fimmu.2020.605958 33384693 PMC7770133

[B72] Nardo G. Iennaco R. Fusi N. Heath P. R. Marino M. Trolese M. C.. (2013). Transcriptomic indices of fast and slow disease progression in two mouse models of amyotrophic lateral sclerosis. Brain 136, 3305–3332. 10.1093/brain/awt250 24065725

[B73] Nardo G. Trolese M. C. Verderio M. Mariani A. De Paola M. Riva N.. (2018). Counteracting roles of MHCI and CD8(+) T cells in the peripheral and central nervous system of ALS SOD1(G93A) mice. Mol. Neurodegener. 13:42. 10.1186/s13024-018-0271-7 30092791 PMC6085701

[B74] O'Brien R. J. Wong P. C. (2011). Amyloid precursor protein processing and Alzheimer's disease. Annu. Rev. Neurosci. 34, 185–204. 10.1146/annurev-neuro-061010-113613 21456963 PMC3174086

[B75] Oliveira A. L. Thams S. Lidman O. Piehl F. Hökfelt T. Kärre K.. (2004). A role for MHC class I molecules in synaptic plasticity and regeneration of neurons after axotomy. Proc. Natl. Acad. Sci. USA. 101, 17843–17848. 10.1073/pnas.0408154101 15591351 PMC539738

[B76] Pan Y. Tian D. Wang H. Zhao Y. Zhang C. Wang S.. (2021). Inhibition of perforin-mediated neurotoxicity attenuates neurological deficits after ischemic stroke. Front. Cell. Neurosci. 15:664312. 10.3389/fncel.2021.664312 34262436 PMC8274971

[B77] Parekh V. V. Wu L. Olivares-Villagómez D. Wilson K. T. Van Kaer L. (2013). Activated invariant NKT cells control central nervous system autoimmunity in a mechanism that involves myeloid-derived suppressor cells. J. Immunol. 190, 1948–1960. 10.4049/jimmunol.1201718 23345328 PMC3577977

[B78] Pasciuto E. Burton O. T. Roca C. P. Lagou V. Rajan W. D. Theys T.. (2020). Microglia require CD4 T cells to complete the fetal-to-adult transition. Cell 182, 625–640.e24. 10.1016/j.cell.2020.06.026 32702313 PMC7427333

[B79] Patel D. Zhang X. Farrell J. J. Chung J. Stein T. D. Lunetta K. L.. (2021). Cell-type-specific expression quantitative trait loci associated with Alzheimer disease in blood and brain tissue. Transl. Psychiatry 11:250. 10.1038/s41398-021-01373-z 33907181 PMC8079392

[B80] Pearce E. L. Shedlock D. J. Shen H. (2004). Functional characterization of MHC class II-restricted CD8+CD4– and CD8–CD4– T cell responses to infection in CD4–/– Mice1. J. Immunol. 173, 2494–2499. 10.4049/jimmunol.173.4.2494 15294964

[B81] Perlmutter L. S. Scott S. A. Barron E. Chui H. C. (1992). MHC class II-positive microglia in human brain: association with Alzheimer lesions. J. Neurosci. Res. 33, 549–558. 10.1002/jnr.490330407 1484388

[B82] Pido-Lopez J. Andre R. Benjamin A. C. Ali N. Farag S. Tabrizi S. J.. (2018). In vivo neutralization of the protagonist role of macrophages during the chronic inflammatory stage of Huntington's disease. Sci. Rep. 8:11447. 10.1038/s41598-018-29792-x 30061661 PMC6065433

[B83] Pishesha N. Harmand T. J. Ploegh H. L. (2022). A guide to antigen processing and presentation. Nat. Rev. Immunol. 22, 751–764. 10.1038/s41577-022-00707-2 35418563

[B84] Priya R. Brutkiewicz R. R. (2020). Brain astrocytes and microglia express functional MR1 molecules that present microbial antigens to mucosal-associated invariant T (MAIT) cells. J. Neuroimmunol. 349:577428. 10.1016/j.jneuroim.2020.577428 33096293 PMC7680413

[B85] Ramagopalan S. V. Ebers G. C. (2009). Multiple sclerosis: major histocompatibility complexity and antigen presentation. Genome Med. 1:105. 10.1186/gm105 19895714 PMC2808740

[B86] Roche P. A. Furuta K. (2015). The ins and outs of MHC class II-mediated antigen processing and presentation. Nat. Rev. Immunol. 15, 203–216. 10.1038/nri3818 25720354 PMC6314495

[B87] Rock K. L. Reits E. Neefjes J. (2016). Present yourself! By MHC class I and MHC class II molecules. Trends Immunol. 37, 724–737. 10.1016/j.it.2016.08.010 27614798 PMC5159193

[B88] Salou M. Nicol B. Garcia A. Baron D. Michel L. Elong-Ngono A.. (2016). Neuropathologic, phenotypic and functional analyses of mucosal associated invariant T cells in multiple sclerosis. Clin. Immunol. 166–167, 1–11. 10.1016/j.clim.2016.03.014 27050759

[B89] Sapp E. Kegel K. B. Aronin N. Hashikawa T. Uchiyama Y. Tohyama K.. (2001). Early and progressive accumulation of reactive microglia in the Huntington disease brain. J. Neuropathol. Exp. Neurol. 60, 161–172. 10.1093/jnen/60.2.161 11273004

[B90] Serriari N. E. Eoche M. Lamotte L. Lion J. Fumery M. Marcelo P.. (2014). Innate mucosal-associated invariant T (MAIT) cells are activated in inflammatory bowel diseases. Clin. Exp. Immunol. 176, 266–274. 10.1111/cei.12277 24450998 PMC3992039

[B91] Shrinivasan R. Wyatt-Johnson S. K. Brutkiewicz R. R. (2024). The MR1/MAIT cell axis in CNS diseases. Brain Behav. Immun. 116, 321–328. 10.1016/j.bbi.2023.12.029 38157945 PMC10842441

[B92] Sieberts S. K. Perumal T. M. Carrasquillo M. M. Allen M. Reddy J. S. Hoffman G. E.. (2020). Large eQTL meta-analysis reveals differing patterns between cerebral cortical and cerebellar brain regions. Sci Data 7:340. 10.1038/s41597-020-00642-8 33046718 PMC7550587

[B93] Simister N. E. Ahouse J. C. (1996). The structure and evolution of FcRn. Res Immunol. 147, 333–337. 10.1016/0923-2494(96)89647-7 8876062

[B94] Smith M. E. (2001). Phagocytic properties of microglia in vitro: implications for a role in multiple sclerosis and EAE. Microsc. Res. Tech. 54, 81–94. 10.1002/jemt.1123 11455615

[B95] Smolders J. Remmerswaal E. B. Schuurman K. G. Melief J. Van Eden C. G. Van Lier R. A.. (2013). Characteristics of differentiated CD8(+) and CD4(+) T cells present in the human brain. Acta Neuropathol. 126, 525–535. 10.1007/s00401-013-1155-0 23880787

[B96] Song S. Miranda C. J. Braun L. Meyer K. Frakes A. E. Ferraiuolo L.. (2016). Major histocompatibility complex class I molecules protect motor neurons from astrocyte-induced toxicity in amyotrophic lateral sclerosis. Nat. Med. 22, 397–403. 10.1038/nm.4052 26928464 PMC4823173

[B97] Sreejit G. Fleetwood A. J. Murphy A. J. Nagareddy P. R. (2020). Origins and diversity of macrophages in health and disease. Clin. Transl. Immunol. 9:e1222. 10.1002/cti2.1222 33363732 PMC7750014

[B98] Steinman R. M. (1988). Cytokines amplify the function of accessory cells. Immunol. Lett. 17, 197–202. 10.1016/0165-2478(88)90028-4 3286486

[B99] Stozicka Z. Zilka N. Novak P. Kovacech B. Bugos O. Novak M.. (2010). Genetic background modifies neurodegeneration and neuroinflammation driven by misfolded human tau protein in rat model of tauopathy: implication for immunomodulatory approach to Alzheimer's disease. J. Neuroinflammation 7:64. 10.1186/1742-2094-7-64 20937161 PMC2958906

[B100] Subbarayan M. S. Hudson C. Moss L. D. Nash K. R. Bickford P. C. (2020). T cell infiltration and upregulation of MHCII in microglia leads to accelerated neuronal loss in an α-synuclein rat model of Parkinson's disease. J. Neuroinflammation 17:242. 10.1186/s12974-020-01911-4 32799878 PMC7429710

[B101] Swain S. L. (1983). T cell subsets and the recognition of MHC class. Immunol. Rev. 74, 129–142. 10.1111/j.1600-065X.1983.tb01087.x 6226585

[B102] Terabe M. Berzofsky J. A. (2014). The immunoregulatory role of type I and type II NKT cells in cancer and other diseases. Cancer Immunol. Immunother. 63, 199–213. 10.1007/s00262-013-1509-4 24384834 PMC4012252

[B103] Tominaga K. Yamagiwa S. Setsu T. Kimura N. Honda H. Kamimura H.. (2017). Possible involvement of mucosal-associated invariant T cells in the progression of inflammatory bowel diseases. Biomed. Res. 38, 111–121. 10.2220/biomedres.38.111 28442662

[B104] Tomiyama A. Cartarozzi L. P. De Oliveira Coser L. Chiarotto G. B. Oliveira A. L. R. (2023). Neuroprotection by upregulation of the major histocompatibility complex class I (MHC I) in SOD1(G93A) mice. Front. Cell. Neurosci. 17:1211486. 10.3389/fncel.2023.1211486 37711512 PMC10498468

[B105] Tooyama I. Kimura H. Akiyama H. Mcgeer P. L. (1990). Reactive microglia express class I and class II major histocompatibility complex antigens in Alzheimer's disease. Brain Res. 523, 273–280. 10.1016/0006-8993(90)91496-4 2400911

[B106] Trager U. Andre R. Lahiri N. Magnusson-Lind A. Weiss A. Grueninger S.. (2014). HTT-lowering reverses Huntington's disease immune dysfunction caused by NFkappaB pathway dysregulation. Brain 137, 819–833. 10.1093/brain/awt355 24459107 PMC3983408

[B107] Treiner E. Duban L. Bahram S. Radosavljevic M. Wanner V. Tilloy F.. (2003). Selection of evolutionarily conserved mucosal-associated invariant T cells by MR1. Nature 422, 164–169. 10.1038/nature01433 12634786

[B108] Unger M. S. Li E. Scharnagl L. Poupardin R. Altendorfer B. Mrowetz H.. (2020). CD8(+) T-cells infiltrate Alzheimer's disease brains and regulate neuronal- and synapse-related gene expression in APP-PS1 transgenic mice. Brain Behav. Immun. 89, 67–86. 10.1016/j.bbi.2020.05.070 32479993

[B109] Vahsen B. F. Nalluru S. Morgan G. R. Farrimond L. Carroll E. Xu Y.. (2023). C9orf72-ALS human iPSC microglia are pro-inflammatory and toxic to co-cultured motor neurons via MMP9. Nat. Commun. 14:5898. 10.1038/s41467-023-41603-0 37736756 PMC10517114

[B110] van den Bosch A. M. R. Van Der Poel M. Fransen N. L. Vincenten M. C. J. Bobeldijk A. M. Jongejan A.. (2024). Profiling of microglia nodules in multiple sclerosis reveals propensity for lesion formation. Nat. Commun. 15:1667. 10.1038/s41467-024-46068-3 38396116 PMC10891081

[B111] van Lith M. Mcewen-Smith R. M. Benham A. M. (2010). HLA-DP, HLA-DQ, and HLA-DR have different requirements for invariant chain and HLA-DM. J. Biol. Chem. 285, 40800–40808. 10.1074/jbc.M110.148155 20959457 PMC3003381

[B112] Wang B. Y. Ye Y. Y. Qian C. Zhang H. B. Mao H. X. Yao L. P.. (2021). Stress increases MHC-I expression in dopaminergic neurons and induces autoimmune activation in Parkinson's disease. Neural. Regen. Res. 16, 2521–2527. 10.4103/1673-5374.313057 33907043 PMC8374590

[B113] Wang Z. K. Xue L. Wang T. Wang X. J. Su Z. Q. (2016). Infiltration of invariant natural killer T cells occur and accelerate brain infarction in permanent ischemic stroke in mice. Neurosci. Lett. 633, 62–68. 10.1016/j.neulet.2016.09.010 27637387

[B114] Watts C. Powis S. (1999). Pathways of antigen processing and presentation. Rev. Immunogenet. 1, 60–74. 11256573

[B115] Wieczorek M. Abualrous E. T. Sticht J. Álvaro-Benito M. Stolzenberg S. Noé F.. (2017). Major histocompatibility complex (MHC) class I and MHC class II proteins: conformational plasticity in antigen presentation. Front. Immunol. 8:292. 10.3389/fimmu.2017.00292 28367149 PMC5355494

[B116] Wilson I. A. Bjorkman P. J. (1998). Unusual MHC-like molecules: CD1, Fc receptor, the hemochromatosis gene product, and viral homologs. Curr. Opin. Immunol. 10, 67–73. 10.1016/S0952-7915(98)80034-4 9523114

[B117] Wyatt-Johnson S. K. Afify R. Brutkiewicz R. R. (2024). The immune system in neurological diseases: what innate-like T cells have to say. J. Allergy Clin. Immunol. 153, 913–923. 10.1016/j.jaci.2024.02.003 38365015 PMC10999338

[B118] Wyatt-Johnson S. K. Brutkiewicz R. R. (2020). The complexity of microglial interactions with innate and adaptive immune cells in Alzheimer's disease. Front. Aging Neurosci. 12:592359. 10.3389/fnagi.2020.592359 33328972 PMC7718034

[B119] Wyatt-Johnson S. K. Kersey H. N. Codocedo J. F. Newell K. L. Landreth G. E. Lamb B. T.. (2023). Control of the temporal development of Alzheimer's disease pathology by the MR1/MAIT cell axis. J. Neuroinflammation 20:78. 10.1186/s12974-023-02761-6 36944969 PMC10029194

[B120] Yewdell J. W. Bennink J. R. (1992). Cell biology of antigen processing and presentation to major histocompatibility complex class I molecule-restricted T lymphocytes. Adv. Immunol. 52, 1–123. 10.1016/S0065-2776(08)60875-5 1442305

[B121] Zalocusky K. A. Najm R. Taubes A. L. Hao Y. Yoon S. Y. Koutsodendris N.. (2021). Neuronal ApoE upregulates MHC-I expression to drive selective neurodegeneration in Alzheimer's disease. Nat. Neurosci. 24, 786–798. 10.1038/s41593-021-00851-3 33958804 PMC9145692

[B122] Zhang Y. Bailey J. T. Xu E. Singh K. Lavaert M. Link V. M.. (2022). Mucosal-associated invariant T cells restrict reactive oxidative damage and preserve meningeal barrier integrity and cognitive function. Nat. Immunol. 23, 1714–1725. 10.1038/s41590-022-01349-1 36411380 PMC10202031

[B123] Zhou Y. Song W. M. Andhey P. S. Swain A. Levy T. Miller K. R.. (2020). Human and mouse single-nucleus transcriptomics reveal TREM2-dependent and TREM2-independent cellular responses in Alzheimer's disease. Nat. Med. 26, 131–142. 10.1038/s41591-019-0695-9 31932797 PMC6980793

